# The complete chloroplast genome sequence of *Vitis yeshanensis* strain ‘SJTU004’

**DOI:** 10.1080/23802359.2019.1674739

**Published:** 2019-10-11

**Authors:** Rongfang Li, Junliang Zhou, Wei Wu, Quanyou Tian, Jiang Lu, Shiren Song

**Affiliations:** a Center for Viticulture and Enology, School of Agriculture and Biology, Shanghai Jiao Tong University, Shanghai, China;; b Institute of Pomological Sciences, Guizhou Academy of Agricultural Sciences, Gui zhou, China

**Keywords:** *Vitis yeshanensis*, chloroplast genome, Illumina sequencing, phylogenetic tree

## Abstract

This study first released the complete chloroplast genome of *Vitis yeshanensis.* The circular genome was 161,188 bp in length, composed of a large single-copy region (LSC, 161,100–89,334 bp) and a small single-copy region (SSC, 115,689–134,745 bp), separated by two inverted repeat regions (IRA, 89,335–115,688 bp; IRB, 134,746–161,099 bp). The genome encoded 134 genes, containing 88 protein-coding genes (PCGs), 38 tRNA genes, and 8 rRNA genes. The phylogenetic tree showed a close relationship between *V. yeshanensis* and *V. amurensis.*

*Vitis yeshanensis*, a hardy native *Vitis* species of China, mainly distributed in Yanshan Mountains (Wan et al. 2008a). This species could also resistant to downy mildew (Wan et al. 2007). But, the study about this species is only descriptive research. For better screening and utilisation of resistant germplasm from *V. yeshanensis*, we sequenced the complete chloroplast genome of one strain ‘SJTU004’ (GenBank: MN205309), and constructed the phylogenetic tree with other *Vitis* species.

Young leaves were collected from ‘SJTU004’ that were planted in the grape germplasm and breeding nursery of Shanghai Jiao Tong University, Shanghai, China (121°26′E; 31°02′N). DNA was extracted by CTAB method described by Fu et al. (2019). Illumina Hiseq paired-end technology was used to sequence the chloroplast genome, and 3.78 Gb high-quality bases were used to assemble the genome by MITObim v1.8 (Hahn et al. 2013) and SOAPdenovo v2.04 (Li et al. 2010). DOGMA was used to predict genes and BLAST 2.6.0+ was used to annotate the genes’ function.

The genome length was 161,188 bp, containing a large single-copy (LSC) region (161,100–89,334 bp) and a small single-copy (SSC) region (115,689–134,745 bp), separated by two inverted repeat regions (IRA, 89335–115,688 bp; IRB, 134,746–161,099 bp). The genome could encode 134 genes, including 88 protein-coding genes (PCGs), 38 tRNA genes, and 8 rRNA genes. Among these genes, 19 genes have one intron (*atpF*, *ndhA*, two *ndhB*, *petB*, *petD*, two *rpl2*, *rpl16*, *rpoc1*, *rps16*, two *tRNA-Ala*, *tRNA-Gly*, *two tRNA-Ile*, *tRNA-Leu*, *tRNA-Lys*, and *tRNA-Val*), four genes have two introns (*clpP*, ycf3, and two *rpl12*), and other genes without intron.

The chloroplast genome sequences of *V. yeshanensis* and other 18 *Vitis* species were aligned online by MAFFT version 7 (Katoh et al. 2017) and were used to construct neighbour-joining (NJ) phylogenetic tree with 1000 bootstrap replications by MEGA X (Kumar et al. 2018). Phylogenetic tree indicated that *V. yeshanensis* was clustered with other East-Asian *Vitis* species, and was closely related to *V. amurensis* (Figure 1). The morphological traits also indicated that *V. yeshanensis* had a close relationship with *V. amurensis* (Wan et al. 2008b). These results suggest that *V. yeshanensis* and *V. amurensis* might have a common origin. The chloroplast genome information of *V. yeshanensis* also provides basic data for further study on germplasm conservation and evolutionary relationships of *Vitis* species of China.

**Figure 1.**
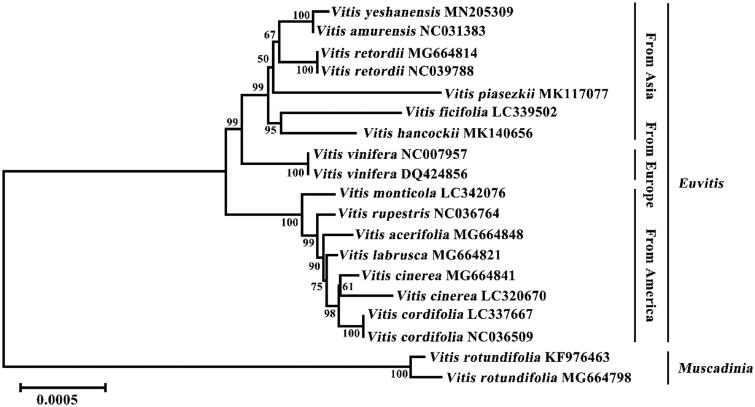
Neighbour-joining phylogenetic tree of 19 *Vitis* species chloroplast genomes (LSC, IRA, and SSC regions). The numbers next to the nodes were bootstrap values based on 1000 replications.
